# Supplementary material for: The HIV-1 Integrase C-Terminal Domain Induces TAR RNA Structural Changes Promoting Tat Binding
Source: Int J Mol Sci. 2022 Nov 8;23(22):13742. doi: 10.3390/ijms232213742 (PMC9692563; doi:10.3390/ijms232213742)
Supplement: Supplementary file 1 [file ijms-23-13742-s001.zip › ijms-1993269-supplementary.pdf]

The HIV-1 Integrase C-Terminal domain induces TAR RNA structural changes promoting Tat binding.

Cecilia Rocchi[1§], Camille Louvat[1§], Adriana Erica Miele[2,3], Julien Batisse[4], Christophe Guillon[1], Lionel Ballut[1], Daniela Lener[5], Matteo Negroni[5], Marc Ruff[4], Patrice Gouet[1] and Francesca Fiorini[1*].

[1] Molecular Microbiology and Structural Biochemistry, MMSB-IBCP, UMR 5086 CNRS University of Lyon, 7 passage du Vercors, 69367, Lyon Cedex 07, France.

[2] Institute of Analytical Sciences, UMR 5280 CNRS UCBL University of Lyon, 5 Rue de la Doua, 69100 Villeurbanne, France. ORCID 0000-0002-4637-2606

[3] Department of Biochemical Sciences, Sapienza University of Rome, P.le Aldo Moro 5, 00185 Rome, Italy.

[4] Chromatin Stability and DNA Mobility, Department of Integrated Structural Biology, IGBMC, CNRS UMR 7104 – Inserm U 158 University of Strasbourg, 1 rue Laurent Fries, 67404, Illkirch Cedex, France.

[5] RNA architecture and reactivity, IBMC, CNRS UPR 9002 University of Strasbourg, 2, Allée Konrad Roentgen, 67084 Strasbourg Cedex, France.

Supplementary Material

Supplementary Figure 1

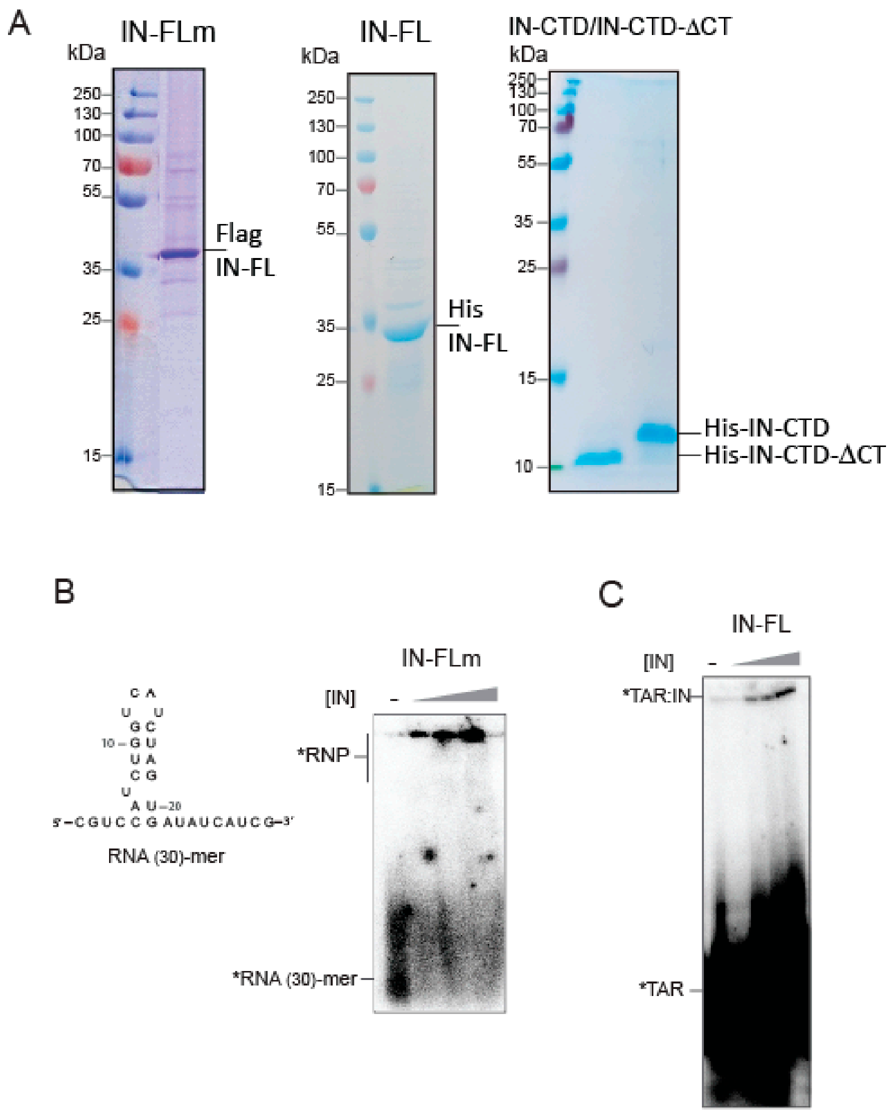

Supplementary Figure S1. A) SDS-PAGE illustrating flag-tagged IN-FL expressed from mammalian expression system, IN-FL, IN-CTD and IN-CTD-ΔCT from *E. coli* used in this study. **B**) Structural model (left panel) of a weakly structured RNA$_{(30)}$-mer used for EMSA assay (right panel) illustrating the interaction of IN-FLm. The RNA substrate was incubated with increasing concentrations of proteins (0; 100; 200; 400 μM) for 30 minutes at 37°C in binding buffer as indicated in experimental procedures. **C**) EMSA assay indicating the binding of IN-FL produced in *E.coli* to TAR substrate.

Supplementary Figure 2

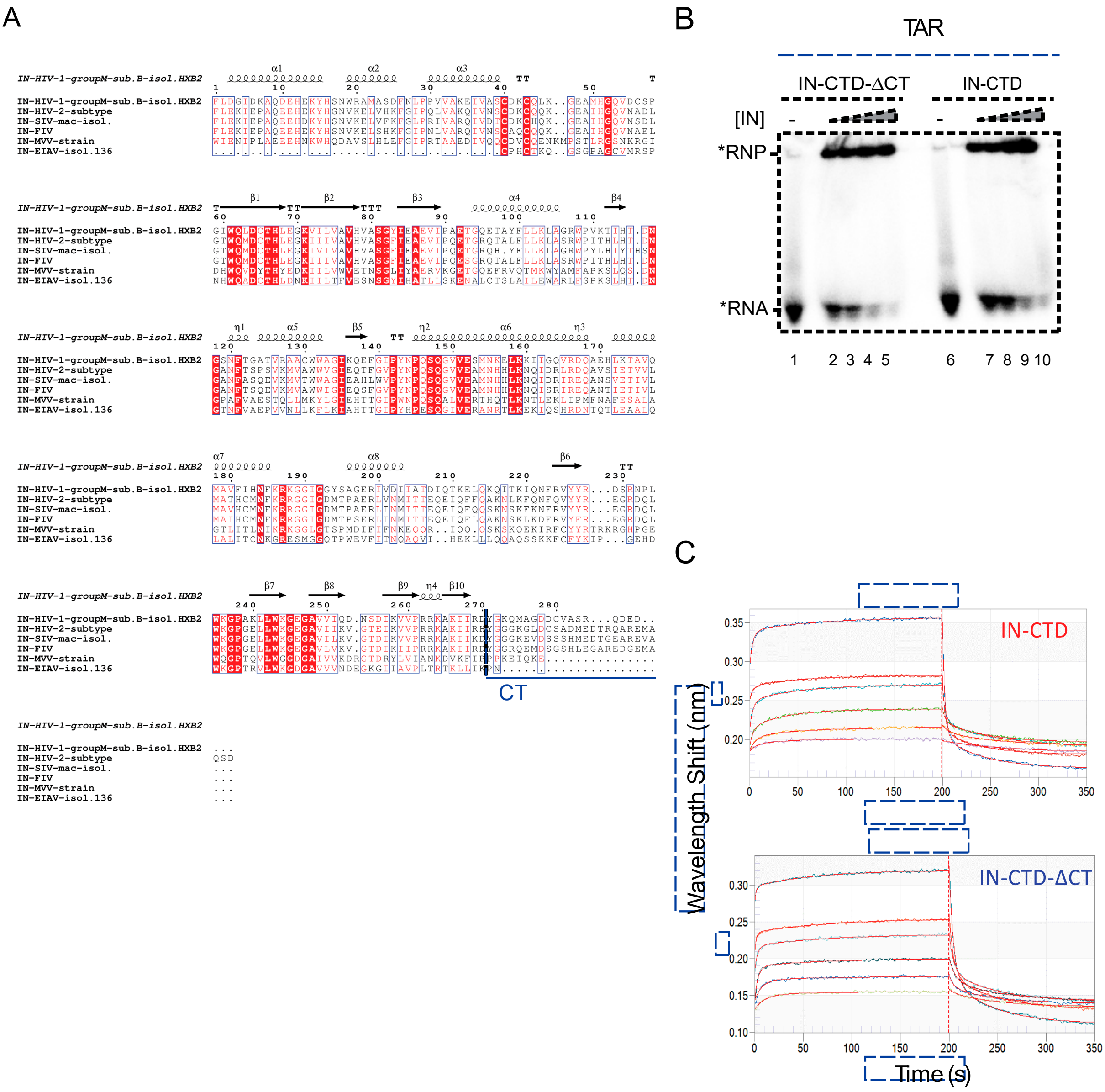

Supplementary Figure S2. A) Sequence alignment of Lentivirus IN proteins. Amino acid sequences were obtained from the HIV database compendium (http://www.hiv.lanl.gov/), aligned using Clustal Omega (https://www.ebi.ac.uk/Tools/msa/clustalo/) and analysed by ESPript 3.0 Web server (65). Secondary structure elements were presented on top of the alignment (helices with squiggles, strands with arrows and turns with TT letters). Red shading indicates sequence identity and boxes indicate sequence similarity, according to physic-chemical properties. HIV - Human Immunodeficiency virus, SIV - *Simian* immunodeficiency virus, FIV - feline immunodeficiency virus, MVV - Maedi visna virus, EIAV - equine infectious anemia virus. **B**) EMSA assay illustrating the interaction of IN-CTD and IN-CTD-ΔCT with TAR

RNA. The RNA substrates (50 nM) are labelled with ^{32}P (black star) and incubated with increasing concentrations of proteins (0; 100, 200, 400 and 800 nM) under the conditions described in 'Materials and Methods' section. **C**) Real-time sensorgrams of the BLI experiment relative to experiment shown in Figure 1F, showing the interaction of IN-CTD and IN-CTD-ΔCT with 5'-biotinylated TAR RNA. Association and dissociation curves are obtained for different protein concentrations 1.8, 2.7, 5.4, 8.1, 16.2 and 32.5 μM, at 37°C in a buffer containing 200 mM NaCl.

Supplementary Figure 3

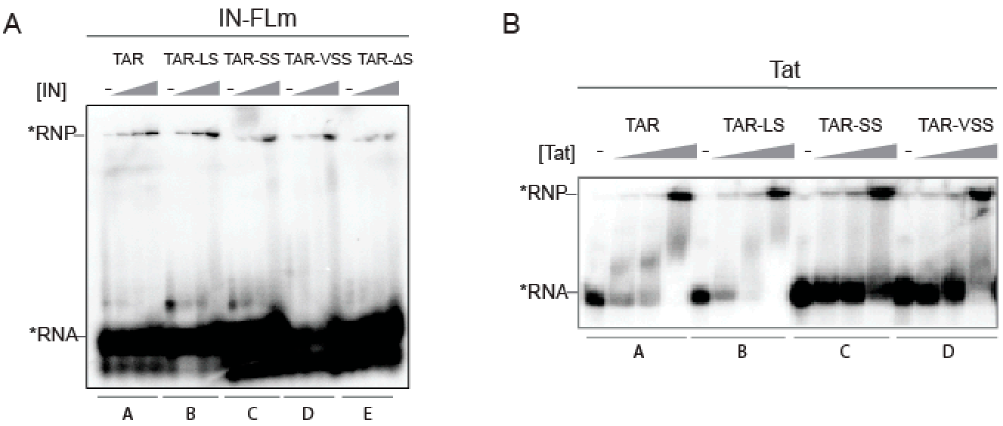

Supplementary Figure S3. A) EMSA assay illustrating the binding of IN-FLm to TAR wild type and mutants. Increasing concentrations of IN-FLm were added to 5'-end radiolabelled TAR RNA and treated as indicated in 'Materials and Methods' paragraph. **B**) EMSA assay showing the interaction of Tat protein (0, 100, 200, 400 nM) with TAR mutants (50 nM) showed in Figure 2A. **C**) Graph showing the fractions of RNA bound by Tat as a function of Tat concentration. Detection, quantification, and data analysis were carried out as described in Figure 2C.

Supplementary Figure 4

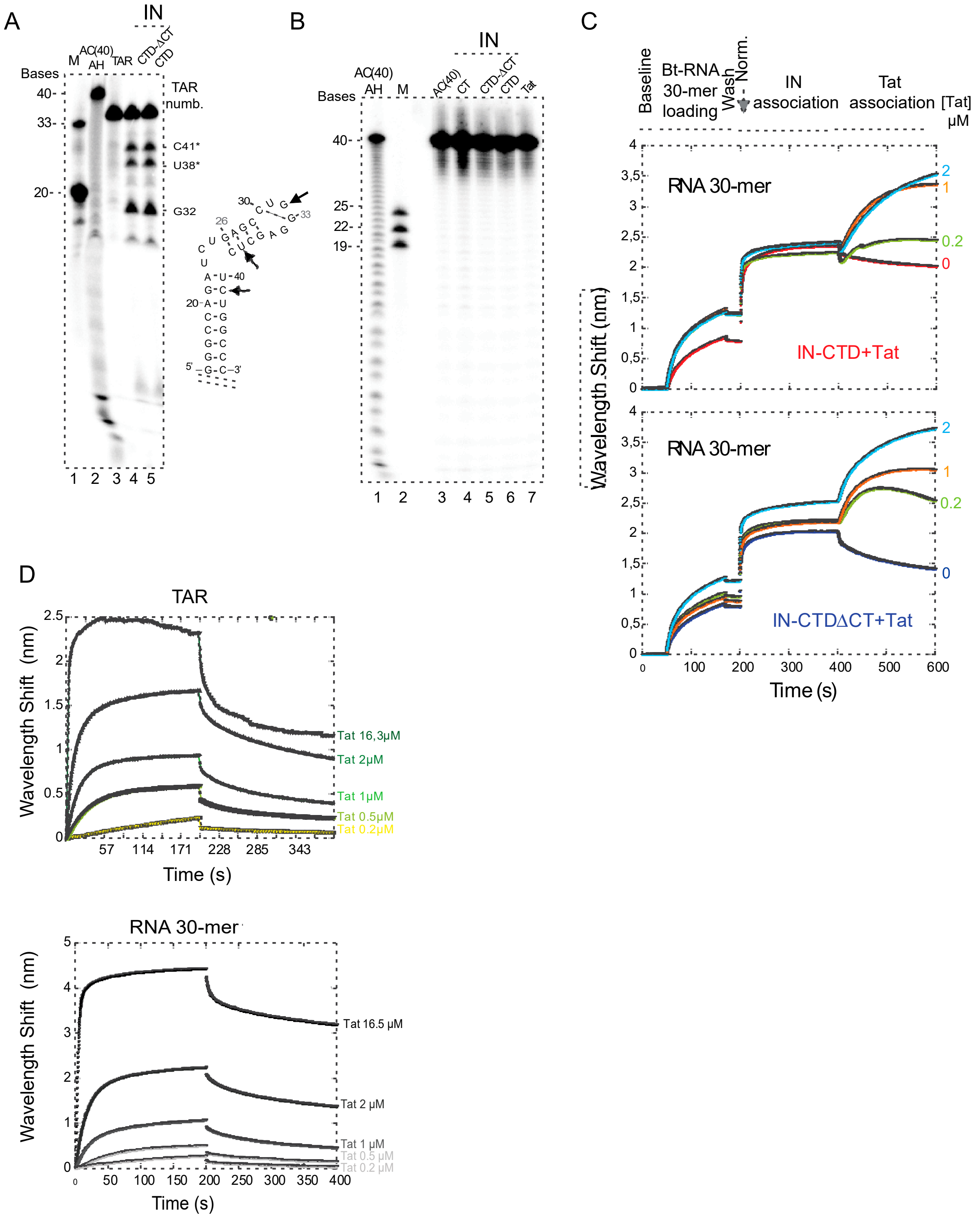

Supplementary Figure S4. A) RNA-denaturing gel showing the effect of IN-CTD and IN-CTD-ΔCT on TAR structure in the absence of RNAse T1. Radiolabelled TAR RNA was incubated with IN truncations in the

same conditions used in Figure 3A. Bands corresponding to spontaneous cleavage in the presence IN-CTD and IN-CTD-ΔCT are identified as position markers. Gel lanes are as follows: (M) Ladder of two RNA transcripts of 33 and 20 nucleotides in length (lane 1); ($AC_{(40)}$-AH) alkaline ladder of $AC_{(40)}$-mer RNA (lane 2); TAR RNA; TAR RNA incubated with IN-CTD-ΔCT (lane 4) and IN-CTD (lane 5). Digestion patterns were mapped on TAR secondary structure depicted on the right of the gel by arrows. **B**) Denaturing RNA gel showing the absence of nucleases in protein samples used for experiments of Figure 3A and Supplementary Figure 4A. The linear $AC_{(40)}$-mer RNA was incubated with indicated protein preparations in the same conditions used for probing experiments: ($AC_{(40)}$-AH) alkaline ladder of $AC_{(40)}$-mer RNA (lane 1); (M) Ladder of three RNA transcripts of 25, 22 and 19 nucleotides in length (lane 2); ($AC_{(40)}$) native $AC_{(40)}$-mer RNA; TAR RNA incubated with IN-CT (lane 4), IN-CTD-ΔCT (lane 5); IN-CTD (lane 5) and Tat (lane 6). **C**) Interferometry sensorgrams of protein-RNA interaction used to calculate the equilibrium dissociation constants of Figure 3E. Bt-$RNA_{(30)}$-mer was first loaded on the streptavidin-coated biosensor for 120 s (Bt-$RNA_{(30)}$-mer loading) then the unbound RNA was washed for 30 s (wash). The sensor was dipped in a solution containing 90 µM of IN protein for 200 s then incubated with different concentrations of Tat (0.2, 1 and 2 µM) for 200 s. **D**) Interferometry sensorgrams to measure the affinity of Tat for immobilized Bt-TAR RNA (upper panel) and Bt-$RNA_{(30)}$-mer (lower panel). The graph shows the real-time association and dissociation kinetics at different Tat concentrations.

Supplementary Figure 5

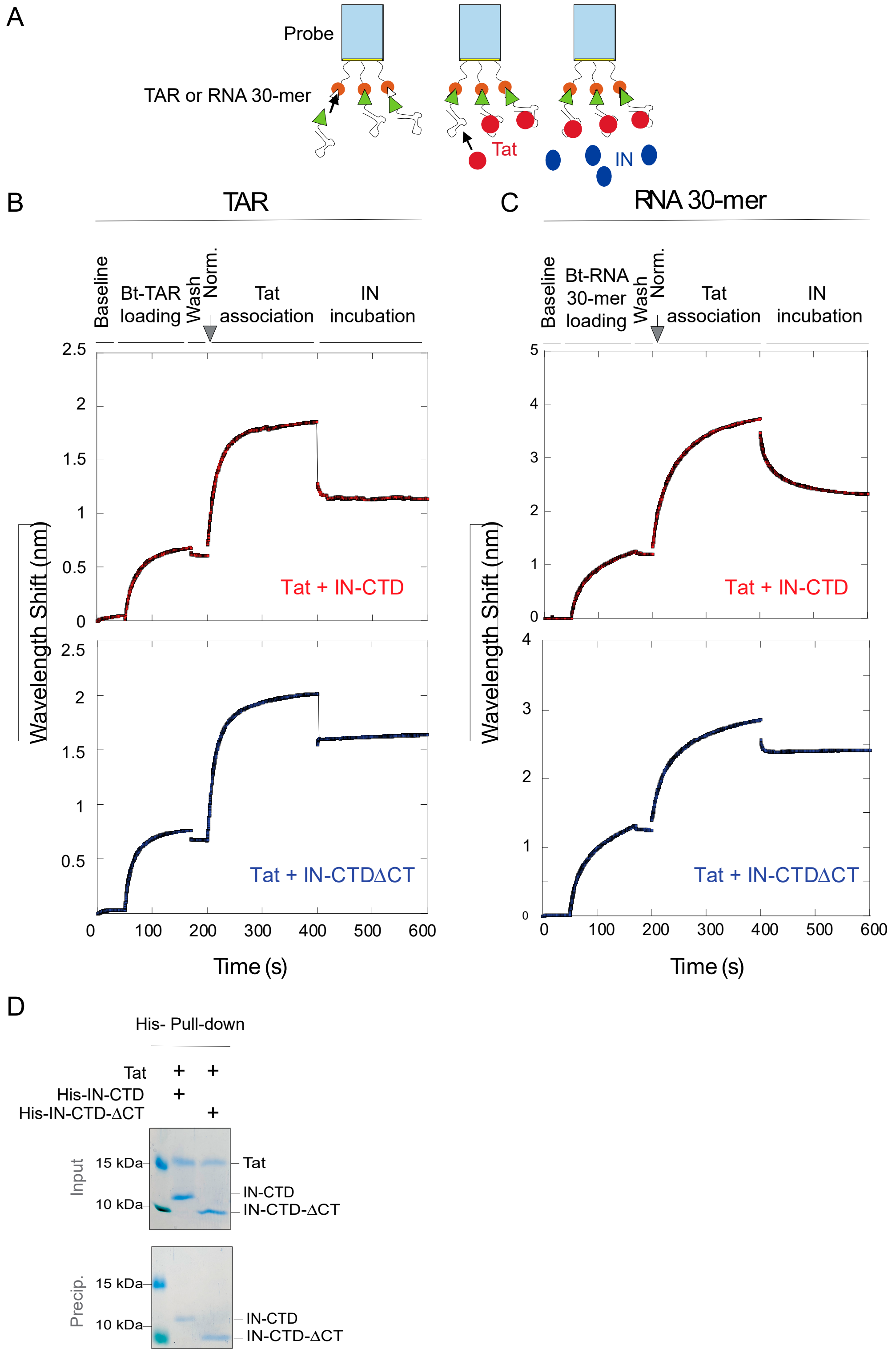

Supplementary Figure S5. A) Schematic illustration of the steps performed during real-time binding experiments by BLI. First, we immobilized Bt-TAR or Bt-RNA$_{(30)}$-mer to streptavidin-coated biosensor and washed out the unbound RNA. Then we soaked the probe in a solution containing Tat. Afterwards, the Tat:RNA bound-biosensor was dipped in a solution containing either IN-CTD or IN-CTD-ΔCT. **B**) Sensorgram showing the association of Tat (32.2 μM) to Bt-TAR (first rising curve between 200 and 400 s). Then the sensor was dipped in a solution containing 32.2 μM of either IN-CTD or IN-CTD-ΔCT for 200 s (curve between 400 and 600 s). **C**) Sensorgrams to measure the association of Tat (2 μM) to Bt-RNA$_{(30)}$-mer (first rising curve between 200 and 400 s). Then the sensor was dipped in a solution containing 90 μM of either IN-CTD or IN-CTD-ΔCT for 200 s (curve between 400 and 600 s). **D**) Histidine pull-down assay using His-tagged IN-CT and IN-CTD-ΔCT. Tagged IN truncations were mixed with Tat and incubated in a buffer containing 200 mM NaCl before co-precipitation. Input (20% of total) and pull-down fractions were analysed by 15% SDS-PAGE followed by Coomassie blue staining.

Supplementary Figure 6

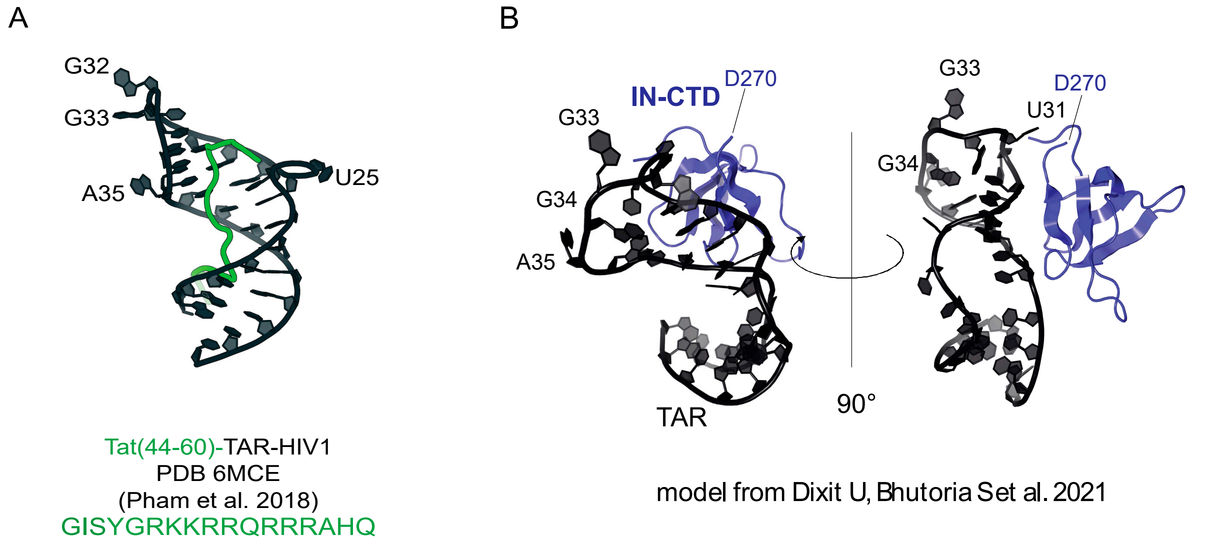

Supplementary Figure S6. A) Solution NMR structure of TAR (black) bound to Tat RBD (green) deposited with PDB code: 6MCE from (Pham et al. 2018). **B**) Structural model of IN-CTD-ΔCT/TAR complex from (Dixit et al. 2021).

Supplementary Table1

**Table S1 RNAs used in this study**		
**Name**	**Sequence (5’-3’)**	**Oligonucleotides and RNA synthesis**
$AG_{(50)}$-mer	gggagagagagagagagagagagagagagagagagagagagagagagaga	FF48/FF54 hybridization
$RNA_{(30)}$-mer	cguccaucuggucaucuagugauaucaucg	chemically synthesized
TAR	gggucucucugguuagaccagaucugagccugggagcucucuggcuaacuagggaaccc	FF90/FF135 hybridization
polyA	gggcacugcuuaagccucaauaaagcuugccuugagugcuucaaguagug	FF155/FF156 – PCR from pNL4-3
DIS	gggcucggcuugcugaagcgcgcacggcaagaggcgag	FF157/FF158 – PCR from pNL4-3
SD/Ψ	gggcgacuggucaguacgccaaaaauuuugacuagcggaggcuagaaggag	FF159/FF160 – PCR from pNL4-3
TAR-LS	gggucucucugguuagaccagaucugagcgcugggacgcucucuggcuaacuagggaac cc	FF91/FF136 hybridization
TAR-SS	gggucucucugguuagaccagaucugagcugggacucucuggcuaacuagggaaccc	FF92/FF137 hybridization
TAR-VSS	Gggucucucugguuagaccagaucugagcgcucucuggcuaacuagggaaccc	FF93/FF138 hybridization
Bt-TAR	gggccagaucugagccugggagcucucuggccc	chemically synthesized
$AC_{(40)}$	acacacacacacacacacacaaaaaaaaaaaaaaaaaaaa	chemically synthesized

Supplementary Table S1. RNA substrates used in this study

Supplementary Table 2

Table S2 DNA Oligonucleotides used to produce RNA substrates	
**Name**	**Sequence (5’-3’)**
FF48	AAATAATACGACTCACTATAGGG
FF54	CTCTCTCTCTCCTCTCTCTCTCTCTCTCTCTCTCTCTCTCTCTCTCCCTATAGTGAGTCGTATTATTT
FF135	AAATAATACGACTCACTATAGGGTCTCTCTGGTTAGACCAGATCTGAGCCTGGGAGCTCTCTGGCTAACTAGGGAACCC
FF90	GGGTTCCCTAGTTAGCCAGAGAGCTCCCAGGCTCAGATCTGGTCTAACCAGAGAGACCCTATAGTGAGTCGTATTATTT
FF136	AAATAATACGACTCACTATAGGGTCTCTCTGGTTAGACCAGATCTGAGCGCTGGGACGCTCTCTGGCTAACTAGGGAACCC
FF91	GGGTTCCCTAGTTAGCCAGAGAGCGTCCCAGCGCTCAGATCTGGTCTAACCAGAGAGACCCTATAGTGAGTCGTATTATTT
FF137	AAATAATACGACTCACTATAGGGTCTCTCTGGTTAGACCAGATCTGAGCTGGGACTCTCTGGCTAACTAGGGAACCC
FF92	GGGTTCCCTAGTTAGCCAGAGAGTCCCAGCTCAGATCTGGTCTAACCAGAGAGACCCTATAGTGAGTCGTATTATTT
FF138	AAATAATACGACTCACTATAGGGTCTCTCTGGTTAGACCAGATCTGAGCGCTCTCTGGCTAACTAGGGAACCC
FF93	GGGTTCCCTAGTTAGCCAGAGAGCGCTCAGATCTGGTCTAACCAGAGAGACCCTATAGTGAGTCGTATTATTT
FF155	AAATAATACGACTCACTATAGGGCACGCAAGCCCAAAAAAGCGCCGAGGCCAAAGAGG
FF156	CCTCTTTGGCCTCGGCGCTTTTTTGGGCTTGCGTGCCCTATAGTGAGTCGTATTATTT
FF157	AAATAATACGACTCACTATAGGGCTCGGCTTGCTGAAGCGCGCACGGCAAGAGGCGAG
FF158	CTCGCCTCTTGCCGTGCGCGCTTCAGCAAGCCGAGCCCTATAGTGAGTCGTATTATTT
FF159	AAATAATACGACTCACTATAGGGCGACTGGTGAGTACGCCAAAAATTTTGACTAGCGGAGGCTAG
FF160	CTAGCCTCCGCTAGTCAAAATTTTTGGCGTACTCACCAGTCGCCCTATAGTGAGTCGTATTATTT

Supplementary Table S2. DNA oligonucleotides used to produce the RNA substrates.

Supplementary References

Dixit U, Bhutoria S, Wu X, Qiu L, Spira M, Mathew S, Harris R, Adams LJ, Cahill S, Pathak R et al. 2021. INI1/SMARCB1 Rpt1 domain mimics TAR RNA in binding to integrase to facilitate HIV-1 replication. *Nat Commun* **12**: 2743.

Pham VV, Salguero C, Khan SN, Meagher JL, Brown WC, Humbert N, de Rocquigny H, Smith JL, D'Souza VM. 2018. HIV-1 Tat interactions with cellular 7SK and viral TAR RNAs identifies dual structural mimicry. *Nat Commun* **9**: 4266.
